# Cystatin B Promotes the Proliferation, Migration, and Invasion of Intrahepatic Cholangiocarcinoma

**DOI:** 10.3390/curroncol32020056

**Published:** 2025-01-21

**Authors:** Dai Zhang, Bao-Ye Sun, Jing-Fang Wu, Zhu-Tao Wang, Su-Su Zheng, Guo-Qiang Sun, Xu-Kang Gao, Jian Zhou, Jia Fan, Bo Hu, Shuang-Jian Qiu, Bo-Heng Zhang

**Affiliations:** 1 Key Laboratory for Carcinogenesis and Cancer Invasion (Ministry of Education), Department of Hepatic Oncology, Liver Cancer Institute, Zhongshan Hospital, Fudan University, 180 Fenglin Road, Shanghai 200032, China; 22111210175@m.fudan.edu.cn (D.Z.); 22111210094@m.fudan.edu.cn (Z.-T.W.); 23111210099@m.fudan.edu.cn (G.-Q.S.); 23211210050@m.fudan.edu.cn (X.-K.G.); 2 Department of Hepatic Oncology, Xiamen Clinical Research Center for Cancer Therapy, Zhongshan Hospital, Fudan University (Xiamen Branch), Xiamen 361015, China; wu.jingfang@zsxmhospital.com (J.-F.W.); zheng.susu@zsxmhospital.com (S.-S.Z.); 3 Department of General Surgery, The Second Xiangya Hospital of Central South University, Changsha 410011, China; 228101038@csu.edu.cn; 4 Department of Liver Surgery and Transplantation, Zhongshan Hospital, Fudan University, Shanghai 200032, China; zhou.jian@zs-hospital.sh.cn (J.Z.); fan.jia@zs-hospital.sh.cn (J.F.); 5 Center for Evidence-Based Medicine, Shanghai Medical School, Fudan University, Shanghai 200032, China

**Keywords:** intrahepatic cholangiocarcinoma, CSTB, proliferation, migration, invasion

## Abstract

**Background and Aims**: Cystatin B (CSTB) has been demonstrated to play a significant role in the pathogenesis of a number of diseases, including the evolution and progression of multiple cancers. Nevertheless, the function of CSTB in intrahepatic cholangiocarcinoma (iCCA) is yet to be fully elucidated. **Methods**: By analyzing transcriptome sequencing data from the FU-iCCA cohort, the iCCA-27 cohort, and three public databases, we identified genes associated with iCCA prognosis and selected CSTB as the subject of our study. The expression of CSTB was examined between tumor tissues and adjacent normal tissues obtained from iCCA patients via Western blot analysis. The clinical significance of CSTB was analyzed through immunohistochemical staining of a tissue microarray. Subsequently, the biological effects of CSTB overexpression or knockdown on iCCA cells were evaluated in vitro and in vivo. **Results**: CSTB expression was markedly elevated in the CCA pathological tissues in comparison to the corresponding adjacent normal tissues. A correlation was identified between higher CSTB expression and poorer patient prognosis in the analysis of 176 iCCA patients. It is noteworthy that overexpression or knockdown experiments demonstrated that CSTB plays a role in the proliferation, migration, and invasion of cells. In subcutaneous tumor models in nude mice, the knockdown of CSTB resulted in smaller tumors in terms of size and weight, and a slower growth rate. **Conclusions**: CSTB plays a significant function in the regulation of iCCA progression and may serve as a promising biomarker for iCCA.

## 1. Introduction

Intrahepatic cholangiocarcinoma (iCCA) is the second-most frequent type of primary liver cancer [1,2,3], with 5-year overall survival (OS) remaining around 9% [4]. Radical resection represents the optimal approach for achieving long-term survival [5]. However, only 30–40% of iCCA patients are suitable for radical resection, with a high postoperative recurrence rate ranging from 40% to 80% [6]. The majority of patients are diagnosed at an advanced stage of the disease [7,8,9]. A deeper understanding of the role of key molecules in iCCA progression may facilitate the development of more effective treatment strategies and contribute to improved patient survival rates.

Cystatin B (CSTB) is an inhibitor of the cysteine protease superfamily [10,11,12], which limits the excessive activity of cysteine proteases by forming tight complexes [13]. Cathepsin belongs to the lysosomal cysteine protease family, also known as cysteine cathepsin, which plays an important role in intracellular proteolysis. Dysregulation of cathepsin is often accompanied by impaired proteolytic activity, which is required in many stages of tumor progression. Imbalance between CSTB and cathepsin is associated with multiple diseases including cancer [13]. Previous studies have found that CSTB plays a significant role in a number of physiological and pathological processes, including protein metabolism, cell proliferation, apoptosis, and tumor metastasis [14,15]. CSTB is aberrantly expressed in a range of neoplastic tissues, including hepatocellular carcinoma [14], pancreatic ductal adenocarcinoma [13], and cervical cancer [16]. Nevertheless, the precise relationship between the aberrant expression of CSTB and the pathogenesis of iCCA remains to be elucidated.

In this study, multiple databases were used to identify genes associated with the prognosis and clinical features of intrahepatic cholangiocarcinoma. Finally, we determined CSTB as our research object. The expression of CSTB in human cancerous and adjacent tissues was investigated. Based on this, we constructed cell lines with CSTB knockdown and overexpression in vitro and investigated the impact of CSTB regulation abnormalities on cell proliferation, migration, and apoptosis using CCK8, scratch, and transwell assays. The objective of this study is to investigate the pivotal function of CSTB in the pathogenesis of intrahepatic cholangiocarcinoma and to identify novel potential therapeutic targets for this disease.

## 2. Materials and Methods

### 2.1. Selection of Patient Cohort

The study included six cohorts of patients. (1) The iCCA-27 cohort: RNA sequencing data of cancer and adjacent tissues from 27 patients with iCCA who underwent radical resection at Zhongshan Hospital between June 2012 and December 2017. (2) The FU-iCCA cohort comprised 244 iCCA patients with complete follow-up data from Zhongshan Hospital, Fudan University [17]. The RNA sequencing data from the surgically resected iCCA samples of this cohort were subjected to analysis. (3) The GSE26566 [18] dataset was downloaded from the Gene Expression Omnibus (GEO) database, including transcriptomes of 104 freshly frozen tumors and 59 matched non-cancerous livers obtained from Australia, Europe, and the United States. (4) The GSE107943 [19] dataset was also obtained from the GEO database and comprised surgical specimens from 30 iCCA patients. Of these, 26 patients had surrounding normal liver tissue available for analysis. (5) The Cholangiocarcinoma dataset from the Cancer Genome Atlas Program (TCGA) comprised 32 tumor tissues and 8 normal tissues. (6) The GSE89749 [20] dataset was downloaded from the GEO database and included integrative genomic and epigenetic analysis of 115 cholangiocarcinoma samples. (7) ZSH cohort: 176 pathologically confirmed iCCA patients who underwent radical resection at Zhongshan Hospital between June 2012 and December 2017. None of the patients included in the study had undergone any form of anticancer treatment prior to surgery. All tumor specimens from the ZSH cohort were fixed with formalin and paraffin-embedded, and subsequently used for tissue microarray (TMA) construction. The ZSH cohort was employed for the purpose of validating the results obtained from the FU-iCCA cohort. Clinical staging was conducted in accordance with the eighth edition of the American Joint Committee on Cancer (AJCC) staging system [21].

### 2.2. Cell Lines

The human iCCA cell lines RBE, HCCC-9810, and CCLP1 were obtained from the Liver Cancer Institute of Fudan University. The RBE and HCCC-9810 cells were cultured in RPMI 1640 medium containing 10% fetal bovine serum at 37 °C in a humidified incubator with 5% CO_2_ in order to facilitate optimal growth. CCLP1 cells were cultured in high-glucose DMEM, supplemented with 10% fetal bovine serum.

### 2.3. RT-qPCR

Total RNA was extracted using the TRIzol method (Invitrogen). Reverse transcription (RT) and quantitative polymerase chain reaction (qPCR) were conducted using Prime Script RT Master Mix and TB Green PCR Kit (Takara, Kyoto, Japan). β-actin was selected as the internal control. The 2^−ΔΔCt^ method was applied to quantify the relative mRNA expression levels of indicated genes [22]. The primers used were as follows: CSTB, 5′-AGGTCCCAGCTTGAAGAGAAA-3′ (F), 5′-CGCAGGTGTACGAAGTCCTC-3′ (R); β-actin, 5′-CACCATTGGCAATGAGCGGTTC-3′ (F), 5′-AGGTCTTTGCGGATGTCCACGT-3′ (R).

### 2.4. Western Blot

Western blot detection was conducted in accordance with the previously described methodology [23]. In summary, the cells were lysed in RIPA lysis buffer, which contained protease and phosphatase inhibitors (EpiZyme, Shanghai, China). The protein samples were separated by 12.5% SDS-PAGE gels, transferred to PVDF membranes, and incubated with primary antibodies and enzyme-labeled secondary antibodies. β-actin was employed as a loading control. An anti-CSTB antibody (1:1000, Cat# ab92449, Abcam, Cambridge, UK) and an anti-β-actin antibody (1:5000, Cat# KC-5A08, KangChen, Shanghai, China) were employed.

### 2.5. Lentivirus-Mediated Construction of Stable Cell Lines

The day prior to transfection, cells were seeded into six-well plates at an inoculation density of approximately 20–30%, with a negative control group included. On the following day, the required virus volume was calculated based on cell density and the MOI (multiplicity of infection) value. Subsequently, the medium in the six-well plates was replaced with 1 mL of medium containing the virus and transfection reagents. After 8–12 h, when changes in cell morphology were observed, the medium was replaced with fresh medium free of viral particles, and the cells were cultured for an additional 24 h. To select successfully transfected cells, culture medium containing 2.5 μg/mL puromycin was used. Once all cells in the negative control group had perished, the medium was replaced with standard culture medium, and the surviving cells were expanded. Finally, qRT-PCR and Western blot analysis were performed to assess the efficiency of gene transfection. The coding sequence of the human CSTB gene was cloned into the lentiviral vector pLVX-CSTB for overexpression by Genechem (Shanghai, China). shCSTB oligonucleotides were cloned into the pLVX-Puro vector (Genechem, Shanghai, China). The sequences were as follows: LV-CSTB-sgRNA (SgCSTB): CCCAGCTTGAAGAGAAAGAAA. The overexpression sgRNA sequence is the CDS section of CSTB.

### 2.6. Assessment of Cell Proliferation Ability

The capacity for cellular proliferation was evaluated through the utilization of the plate colony formation assay and the Cell Counting Kit-8 (CCK-8). The cells were treated in accordance with the designated protocols and subsequently plated into 6-well plates at a density of approximately 1000 cells per well. Following a two-week culture period, the cells were fixed with formaldehyde, stained with crystal violet, and photographed. A total of 10 mL/well of CCK-8 reagent (Bioagrio, Shanghai, China) was added to a 96-well plate containing 2000 cells per well and incubated at 37 °C for 2 h. The optical density values at 450 nm (OD450) were measured with a 96-well multimode plate reader (SpectraMaX iD3, Molecular Devices, San Jose, CA, USA).

### 2.7. Evaluation of Cell Invasion Ability

The capacity of the cells to invade other tissues was evaluated using transwell chambers with 8 mm pore size polycarbonate membrane filters. Following a 24 h transfection period, cells were introduced to the upper chambers of Matrigel-coated (Matrigel Matrix, Corning, NY, USA) membrane-based transwell inserts and cultured for an additional 24 h. For the wound healing (scratch) assay, cells were seeded in 6-well plates. Once the cells had reached confluence, a scratch was made in the monolayer, and digital images were acquired at 0, 24, and 48 h.

### 2.8. Apoptosis Analyses

The status of cell apoptosis was then evaluated using apoptosis kits (Biolegend, Shanghai, China). The cells were resuspended in 400 μL of Annexin V binding buffer and incubated with 5 μL of APC Annexin V and 10 μL of Propidium Iodide Solution at room temperature (25 °C) in the dark for 15 min. Subsequently, the samples were analyzed using flow cytometry (BD FACS Aria II, Piscataway, NJ, USA).

### 2.9. Animal Care and Use

Animal experiments were carried out in accordance with the protocols approved by the Institutional Animal Care and Use Committee of Zhongshan Hospital, Fudan University. Six-week-old nude mice were purchased from Shanghai Jihui. All these mice were housed under specific pathogen-free conditions at the Animal Center of Fudan University.

### 2.10. Subcutaneous Xenograft Tumor Model

A suspension of 1 × 10^7^ cells in 200 μL of PBS was injected subcutaneously into the left flank of six-week-old male nude mice [24,25]. Using six nude mice in each group, tumors were observed on day 14 after subcutaneous injection and regular measurements were initiated. Tumor volume was determined by caliper measurement at 3- to 4-day intervals, with calculations performed according to the formula length (mm) × width^2^ (mm)/2. At the conclusion of the experiment, the mice were euthanized, and the tumors were excised for further analysis.

### 2.11. Tissue Microarray (TMA) and Immunohistochemistry (IHC) Staining

In the ZSH cohort, TMAs containing 176 ICC specimens were constructed. Tissue sections were subjected to immunohistochemical staining in accordance with previously established protocols [26]. Following the blocking of endogenous peroxidase and the retrieval of antigens, the slides were incubated with primary antibody CSTB (1:900, Abcam, Cambridge, UK). Quantitative analysis of tissue microarray immunohistochemical staining intensity was performed using the open-source software QuPath (v0.5.1) [27]. The IHC staining scores (Histochemistry score, H-score) were obtained. The software-generated scoring results were then verified by two pathologists. Subsequently, the relative CSTB expression was calculated by dividing the H-score of cancer and adjacent tissues by the H-score of normal liver tissue.

### 2.12. RNA-Seq

The TRIzol method was used to extract the total RNA of CCLP1 empty vector and shCSTB cells according to the manufacturer’s protocol. RNA-Seq (*n* = 3) was performed on an Illumina Novaseq platform. The transcriptome sequencing and analysis were conducted by Origingene, Shanghai. The raw RNA-seq transcripts were normalized in fragments per kilobase of transcript per million mapped reads (FPKM) and used for pathway enrichment analysis.

### 2.13. Statistical Analysis

The statistical analyses were conducted using R version 4.1.2 and GraphPad 8.0 software. Student’s *t*-test or the Mann–Whitney test was employed to ascertain the existence of statistically significant differences between the continuous variables of the two groups under investigation. In the event of non-homogeneity of variance within the two groups, a non-parametric Wilcoxon rank-sum test was employed. The prognostic value of CSTB was evaluated through Kaplan–Meier survival analyses and log-rank tests, utilizing the R packages survival and survminer. A *p*-value of less than 0.05 was considered to be statistically significant.

## 3. Results

### 3.1. Selection of Molecules Associated with Prognosis and Clinical Features of Intrahepatic Cholangiocarcinoma

Firstly, we collected tumor and adjacent non-tumor tissues from 27 iCCA patients at Zhongshan Hospital, Fudan University, and performed transcriptome sequencing. Based on the transcriptome sequencing data, we identified 6683 genes with higher expression in tumor tissues compared to adjacent tissues (logFC > 1, *p* < 0.05). Subsequently, we screened these genes to identify those that also showed higher expression in tumor tissues in the TCGA cholangiocarcinoma dataset and two GEO cholangiocarcinoma datasets (GSE26566 and GSE107943) (logFC > 1, *p* < 0.05). A total of 608 genes were identified.

**Figure 1:**
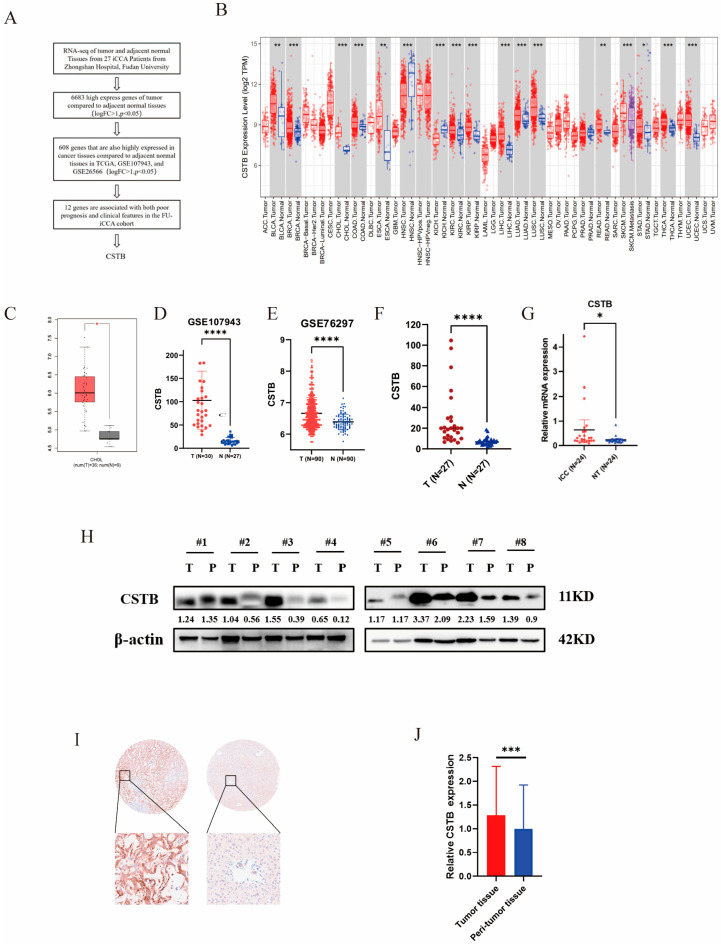
Gene screening process and elevated CSTB expression of malignant cells. (**A**) Gene screening process. (**B**) The expression of CSTB in pan-cancer. (**C**) Comparison of CSTB mRNA expression in cancer tissues and normal tissues in TCGA, GSE107943 (**D**), GSE76297 (**E**), sequencing data of 27 pairs of cancer and adjacent tissues (**F**), 24 pairs of cancer and adjacent tissues (**G**). (**H**) Comparison of CSTB protein expression in iCCA and adjacent tissues. (**I**) Representative images of immunohistochemical expression of CSTB in tumor and adjacent tissue microarrays (**J**) Comparison of expression of CSTB in immunohistochemistry of cancer and adjacent tissue microarrays from 176 patients. * *p* < 0.1; ** *p* < 0.01; *** *p* < 0.001; **** *p* < 0.0001; TCGA, The Cancer Genome Atlas; iCCA, intrahepatic cholangiocarcinoma. The original Western blot figures can be found in Supplementary File S1.

Next, in the FU-iCCA cohort, we screened for genes associated with poor prognosis and clinical features. The screening criteria were as follows: independent prognostic significance (HR > 1, *p* < 0.05), AUC > 0.65, Kaplan–Meier (KM) and Cox survival analyses both *p* < 0.01, and association with four clinical features: TNM stage, vascular invasion, nerve invasion, and regional lymph node metastasis (*p* < 0.05) (Figure 1A).

From this analysis, we identified 12 candidate genes (Figure 1A). Finally, after a review of the literature, CSTB was selected as the focus of our study.

### 3.2. CSTB Is Highly Expressed in Intrahepatic Cholangiocarcinoma and Associated with Poor Clinical Prognosis

**Figure 2:**
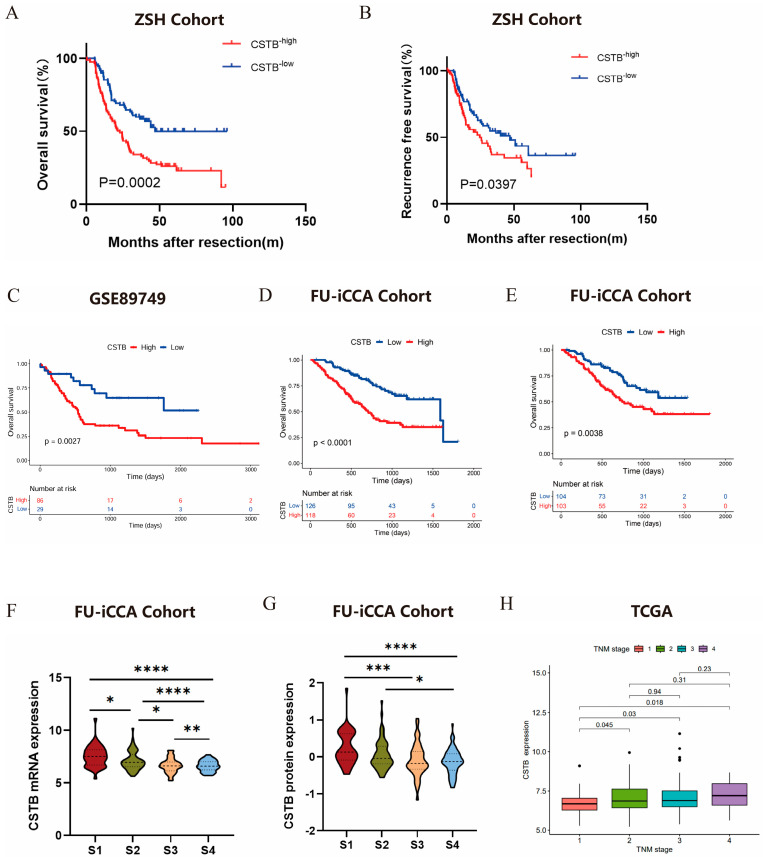
Expression pattern and prognostic value of CSTB in iCCA. Kaplan–Meier survival curves for OS (**A**) and RFS (**B**) of 176 iCCA patients after curative resection according to CSTB expression level in the ZSH cohort. Kaplan–Meier survival curves for OS of cholangiocarcinoma patients after curative resection according to CSTB expression level in the GSE89749 (**C**) High-CSTB mRNA (**D**) or CSTB protein (**E**) expression was significantly associated with poor OS in the FU-iCCA cohort. Comparison of CSTB mRNA expression (**F**) and CSTB protein expression (**G**) across four molecular subtypes identified in the FU-iCCA cohort. (**H**) CSTB mRNA expression in patients with different TNM stages in TCGA * *p* < 0.1; ** *p* < 0.01; *** *p* < 0.001; **** *p* < 0.0001; OS, overall survival; iCCA, intrahepatic cholangiocarcinoma. WB, Western blot; * *p* < 0.05, ** *p* < 0.01, *** *p* < 0.001.

We detected high and low expressions of CSTB in cancer and adjacent tissues in the TCGA pan-cancer data, and found that CSTB is abnormally expressed in various tumors (Figure 1B). Figure 1C–F show the expression of CSTB in tumor and adjacent non-tumor tissues based on TCGA (*p* < 0.05), GSE107943 (*p* < 0.00001), GSE76297 (*p* < 0.00001), and RNA sequencing data from 27 pairs of iCCA patient samples (*p* < 0.00001). To validate these findings, we collected tumor and adjacent tissues from an additional 24 iCCA patients, extracted the RNA, and conducted PCR experiments, which yielded consistent results (*p* < 0.05) (Figure 1G). We also detected CSTB expression in cancer and adjacent tissues of eight iCCA patients by Western blot (WB) and found that in most cases, CSTB expression was higher in cancer tissues than in adjacent tissues (Figure 1H). We then examined the expression levels of CSTB in tumor tissues and adjacent tissues from 176 iCCA patients of the ZSH cohort using immunohistochemistry (IHC) staining (Figure 1I, J). Patients in the ZSH cohort were divided into high-expression and low-expression groups based on CSTB expression levels in tumor tissues. Kaplan–Meier analysis showed that iCCA patients with high CSTB expression had poorer RFS (*p* = 0.0444, Figure 2A) and OS (*p* < 0.0001, Figure 2B). We verified in the GSE89749 dataset that patients with high CSTB expression had lower OS (*p* = 0.0027, Figure 2C) In the FU-iCCA cohort, patients were divided into two groups based on CSTB mRNA expression and protein expression levels, and high mRNA and protein levels of CSTB were associated with poorer overall survival (OS) (*p* < 0.0001 and *p* = 0.0038, Figure 2D,E). In addition, CSTB mRNA and protein levels varied across the four previously defined iCCA molecular subtypes [17], with the lowest levels in subtype S4, which had the best prognosis (Figure 2F,G). Next, we analyzed the cholangiocarcinoma data from TCGA and stratified patients into four groups by TNM stage, with higher expression of CSTB mRNA in the later stages (Figure 2H).

### 3.3. CSTB Promotes Intrahepatic Cholangiocarcinoma Proliferation and Colony Formation

**Figure 3:**
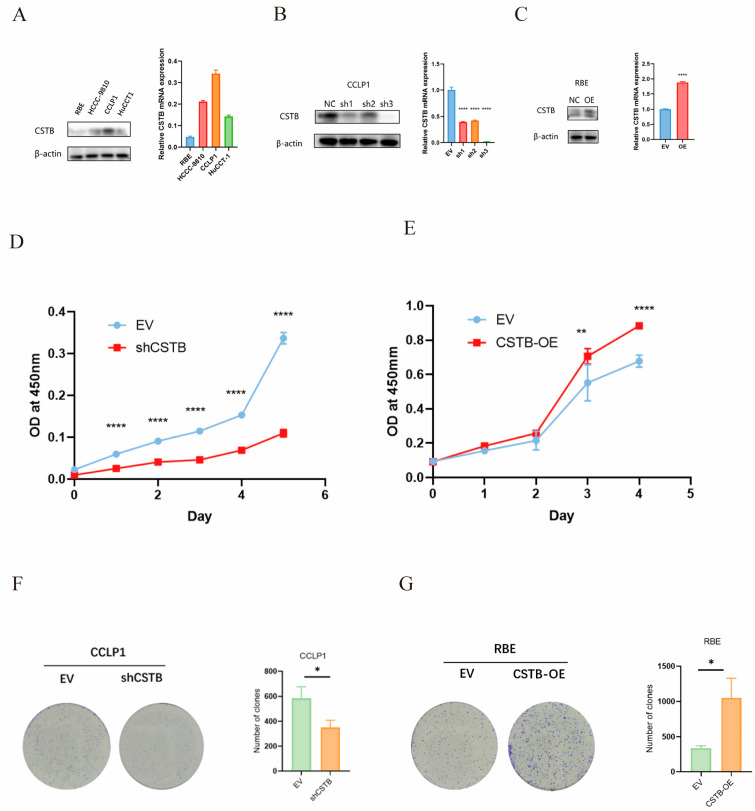
CSTB promotes the multiplication of intrahepatic cholangiocarcinoma (iCCA) cells. (**A**) The expression of CSTB in four wild-type cells. The efficiency of knockdown CSTB (**B**) and overexpression CSTB (**C**) was verified at the protein and mRNA levels. According to the CCK8 detection (**D**,**E**) and colony formation assay (**F**,**G**), it was found that the knockdown of CSTB inhibited the proliferation of iCCA cells, and the overexpression of CSTB promoted the proliferation of iCCA cells. * *p* < 0.05, ** *p* < 0.01, **** *p* < 0.0001. The original Western blot figures can be found in Supplementary File S1.

In light of the unfavorable prognosis of CSTB and its potential carcinogenic effects, we initiated an investigation into the biological function of CSTB in iCCA. In vitro functional experiments were conducted using iCCA cell lines. The expression of CSTB was detected in four intrahepatic cholangiocarcinoma cell lines, including RBE, HCCC9810, CLLP1, and HUCCT-1. The highest levels of CSTB mRNA and protein expression were observed in CCLP1, while the lowest levels were observed in RBE (Figure 3A). RBE cells were transfected with CSTB overexpression lentiviral vectors (RBE-CSTB-OE), and empty lentiviral vectors served as a negative control (RBE-EV). The transfection efficiency was verified by Western blot (WB) and quantitative polymerase chain reaction (qPCR) analysis (Figure 3B). Three lentiviral vectors were employed to suppress CSTB expression in CCLP1 cells. The transfection effects were detected by WB and qPCR (Figure 3C). The cell lines with the best knockdown efficiency were selected for the following experiment. CCK8 and colony formation experiments demonstrated that iCCA cell proliferation was accelerated when CSTB was overexpressed, whereas iCCA cell growth was inhibited when CSTB was silenced (Figure 3D–G). These results collectively substantiated the hypothesis that CSTB promotes the proliferation of iCCA cells.

### 3.4. CSTB Enhances the Migration and Invasion Abilities of iCCA and Inhibits Apoptosis

**Figure 4:**
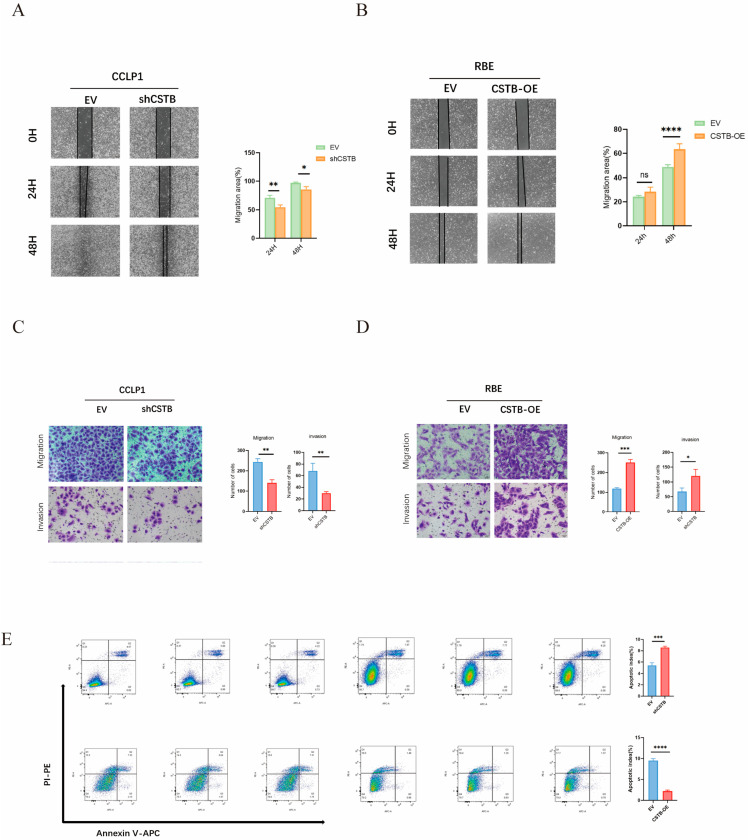
CSTB promotes intrahepatic cholangiocarcinoma (iCCA) cell migration and invasion while decreasing intrahepatic cholangiocarcinoma (iCCA) cell apoptosis. According to the scratch healing assays (**A**,**B**) and migration assay (**C**,**D**), it was found that knockdown of CSTB inhibited the migration ability of iCCA cells, and the overexpression of CSTB promoted the migration ability of iCCA cells. (**C**,**D**) Cell migration and invasion abilities analyzed by the transwell migration assay (20×). (**E**) Flow cytometry analysis performed to assess the apoptosis in different groups of iCCA cells. * *p* < 0.05, ** *p* < 0.01, *** *p* < 0.001, **** *p* < 0.0001, ns: Not significant.

Furthermore, the capacity of iCCA cells to migrate was augmented by the overexpression of CSTB, whereas it was diminished by the silencing of CSTB, as evidenced by the outcomes of wound healing assays (Figure 4A,B). Migration and invasion assays demonstrated that silencing CSTB markedly suppressed the capacity of iCCA cells to migrate and invade, whereas overexpression of CSTB markedly enhanced this capacity (Figure 4C,D). Following the knockdown of CSTB, there was an increase in the apoptosis of intrahepatic cholangiocarcinoma cells. Conversely, the overexpression of CSTB was observed to result in a reduction in the apoptosis of intrahepatic cholangiocarcinoma cells (Figure 4E).

### 3.5. CSTB Plays a Role in the Growth of iCCA In Vivo

**Figure 5:**
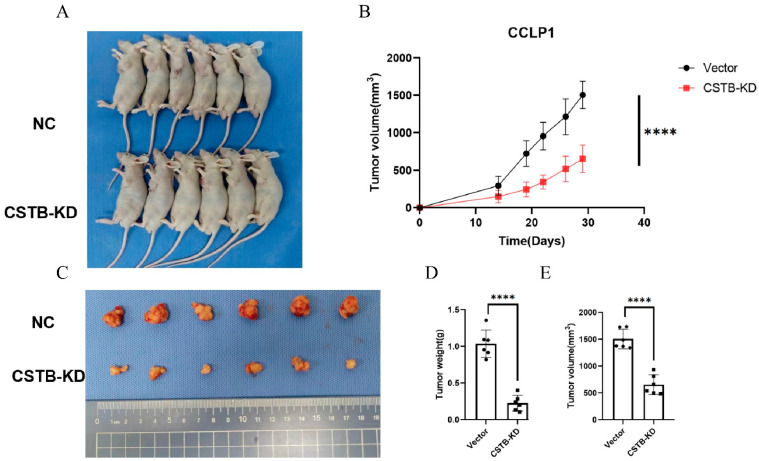
Silencing CSTB inhibited tumor growth in vivo. (**A**) Establishment of the subcutaneous xenograft model with nude mice using an empty vector or CSTB knockdown CCLP1 cells. (**B**) Tumor growth curve representing each group of mice. (**C**) Tumors captured from distinct groups of mice. (**D**,**E**) Analysis of tumor volumes and weight among mice conducted through statistical means. **** *p* < 0.0001.

CCLP1 cells with CSTB knockdown and control cells were subcutaneously injected into nude mice. Tumor growth was observed to be more gradual in the CSTB knockdown group in comparison to the control group (Figure 5A–C,E). Tumor volume was calculated on a three-to-four-day basis (Figure 5B). The mean tumor weight was significantly lower in the CSTB knockdown group than in the control group (Figure 5D).

### 3.6. Exploration of the Mechanism by Which CSTB Promotes iCCA Progression

**Figure 6:**
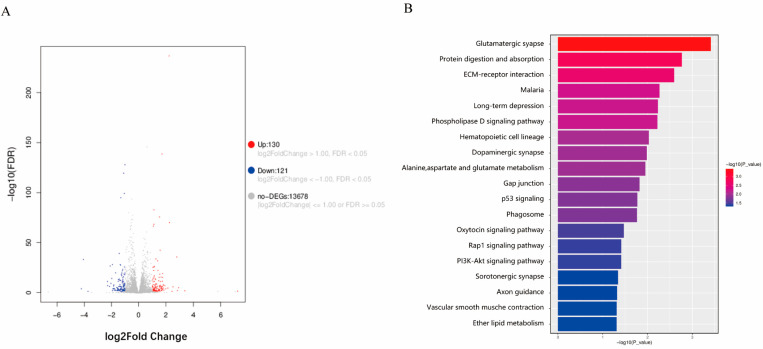
Transcriptome sequencing analysis results of the CCLP1 control group and knockdown group. (**A**) Differentially expressed genes in CCLP1-shCSTB compared to CCLP1-EV. (**B**) Kyoto Encyclopedia of Genes and Genomes (KEGG) analyses of the differentially expressed genes.

To further explore the specific mechanisms by which CSTB affects iCCA progression, we performed transcriptome sequencing on CCLP1-EV and CCLP1-shCSTB cells. A total of 130 upregulated genes and 121 downregulated genes were identified (log2FC > 1.00, FDR < 0.05) (Figure 6A). Kyoto Encyclopedia of Genes and Genomes analyses of the differentially expressed genes revealed that differentially expressed genes are enriched in pathways such as the “p53 signaling pathway” and the “PI3K-Akt signaling pathway” (Figure 6B).

## 4. Discussion

In light of the increasing prevalence of intrahepatic cholangiocarcinoma globally and the fact that the majority of patients are diagnosed at an advanced stage [28], the identification of prognostic factors for the onset and progression of intrahepatic cholangiocarcinoma is of paramount importance [29]. At present, the biomarkers employed in clinical practice include carcinoembryonic antigen (CEA) and carbohydrate antigen 19-9 (CA19-9) [30,31,32]. However, due to their inadequate sensitivity and specificity, their utilization in diagnosing or monitoring iCCA is constrained [33]. Consequently, the identification of novel prognostic markers for intrahepatic cholangiocarcinoma is of paramount importance.

Through the analysis of multiple databases and transcriptome sequencing data, we determined CSTB as our research object. Through the analysis of database data and transcriptome sequencing data, we found that the expression level of CSTB in iCCA tissues was significantly increased compared with adjacent normal tissues. Moreover, the expression level of CSTB was related to the adverse prognostic outcome of iCCA patients, and a high level of CSTB was also associated with a poorer stage of prognosis in iCCA patients. The results of in vitro experiments showed that the knockdown of CSTB significantly hindered the migration, invasion, and proliferation of iCCA cells, and the overexpression of CSTB promoted the migration, invasion, and proliferation of iCCA cells. In vivo experiments demonstrated that the knockdown of CSTB reduced tumor volume, tumor weight, and growth rate. This finding is consistent with other studies; for example, it was observed that the inhibition of CSTB expression in HCC cell lines resulted in restricted cell growth [14].

CSTB functions as an inhibitor of cysteine proteases, particularly cathepsins, which are enzymes involved in the degradation of proteins within lysosomes. By regulating the activity of these proteases, CSTB plays a crucial role in maintaining cellular homeostasis and preventing excessive protein degradation [34]. Some previous studies have explored the specific mechanism by which CSTB affects tumor progression. In pancreatic ductal adenocarcinoma, CSTB competes with cystatin C (CSTC) to bind cathepsin B (CTSB), but CSTC has stronger cathepsin inhibitory activity. The high expression of CSTB in pancreatic ductal adenocarcinoma reduces the inhibition of CTSB, thereby acting as a tumor-promoting factor [13]. In epithelial ovarian cancer, elevated CSTB expression in ovarian tissue indicates tumor progression. The dysregulation of the TGF-β signaling pathway is considered crucial for the development of epithelial ovarian cancer (EOC). It was discovered that the inhibition of TGF-β signaling leads to the abolishment of CSTB suppression, suggesting that the regulation of CSTB is mediated through the TGF-β signaling pathway [10]. We also performed transcriptome sequencing on CCLP1-EV and CCLP1-shCSTB and found that the differentially expressed genes were enriched in pathways such as the “p53 signaling pathway” and the “PI3K-Akt signaling pathway”, However, these results still need to be validated in iCCA.

In addition to the aforementioned results, it is important to acknowledge the limitations of this study. The FU-iCCA and ZSH cohorts were derived from a single medical institution in China. It would be beneficial to conduct external validation of the findings in other iCCA cohorts in order to confirm the generalizability of the results. Furthermore, the functional mechanisms by which CSTB exerts its effects in iCCA cells require further investigation. This should include an examination of potential downstream signaling pathways and the interaction with other molecules.

## 5. Conclusions

In conclusion, the results of our study provide compelling evidence to substantiate the oncogenic function of CSTB in iCCA. This finding is of considerable significance, as it offers new insights into the development and progression of this disease. Monitoring the level of CSTB expression may serve as a valuable prognostic marker. The identification of patients with high CSTB expression may enable healthcare providers to more accurately assess prognosis and develop more targeted treatment strategies.

## Data Availability

All data generated during this study are available from the corresponding author upon reasonable request.

## Supplementary Materials

The following supporting information can be downloaded at: https://www.mdpi.com/article/10.3390/curroncol32020056/s1, File S1: The original Western blot figures.

## Abbreviations

iCCA, intrahepatic cholangiocarcinoma; CSTB, Cystatin B; OS, overall survival; RFS, recurrence-free survival; CEA, carcinoembryonic antigen; CA19-9, carbohydrate antigen 19-9.
