# Supplementary material for: Cystatin B Promotes the Proliferation, Migration, and Invasion of Intrahepatic Cholangiocarcinoma
Source: Curr Oncol. 2025 Jan 21;32(2):56. doi: 10.3390/curroncol32020056 (PMC11854580; doi:10.3390/curroncol32020056)

Figure 2A

Wild type actin

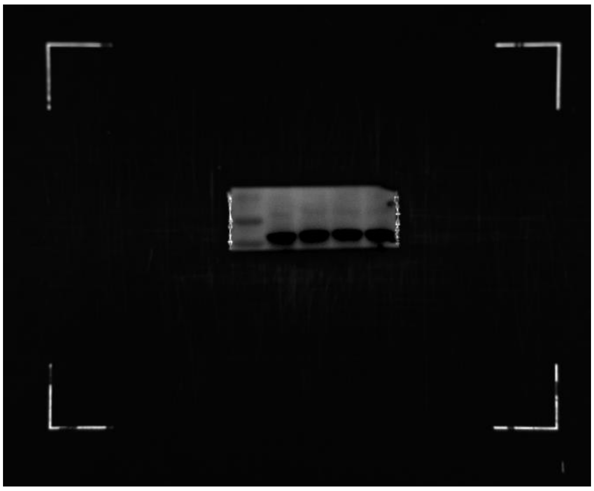

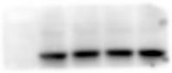

Wild type CSTB

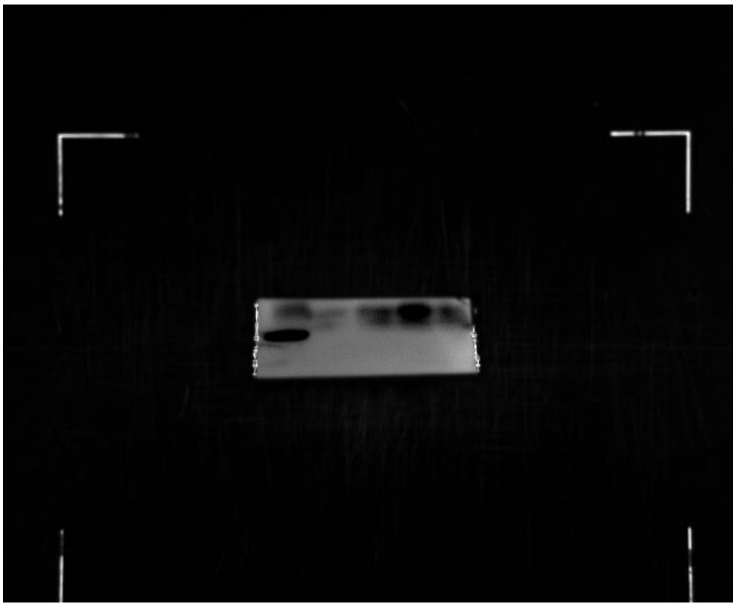

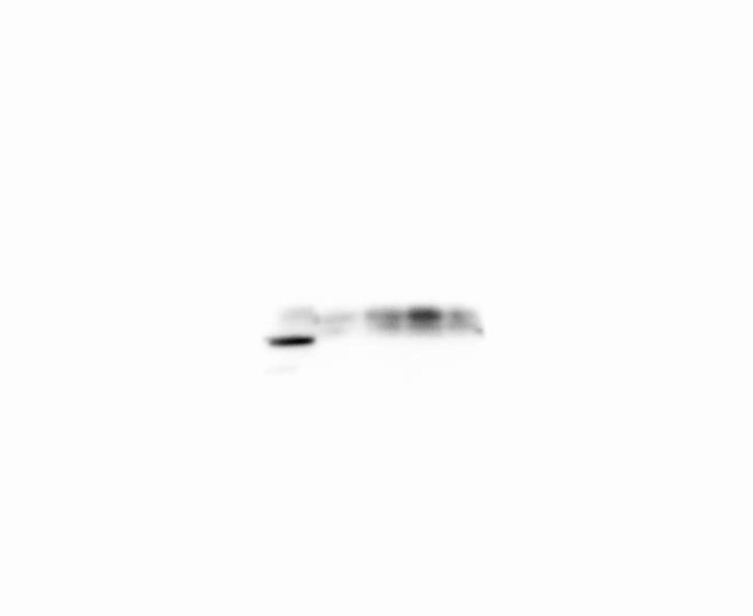

Figure 2B

Ev and shCSTB actin

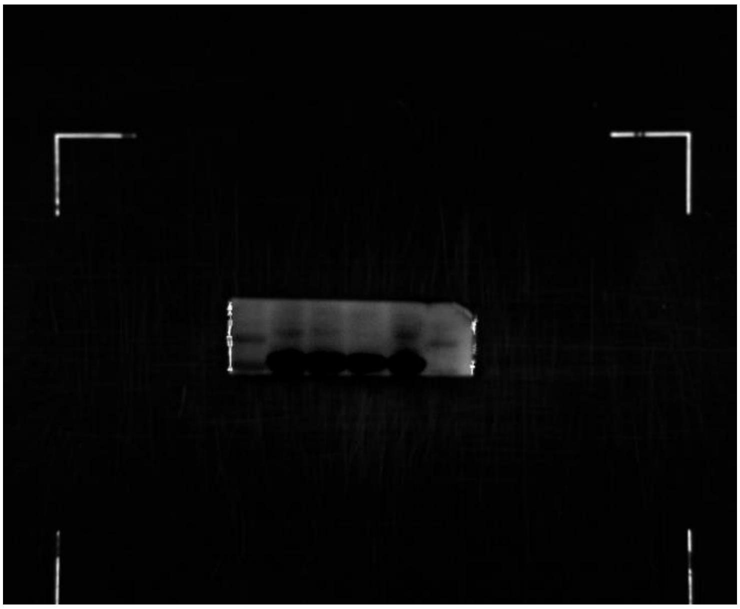

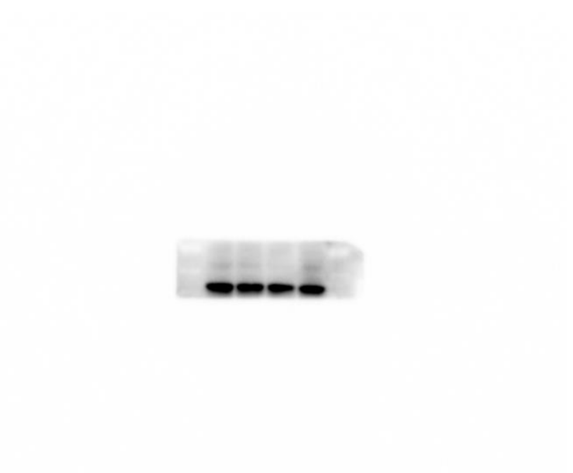

Ev and shCSTB CSTB

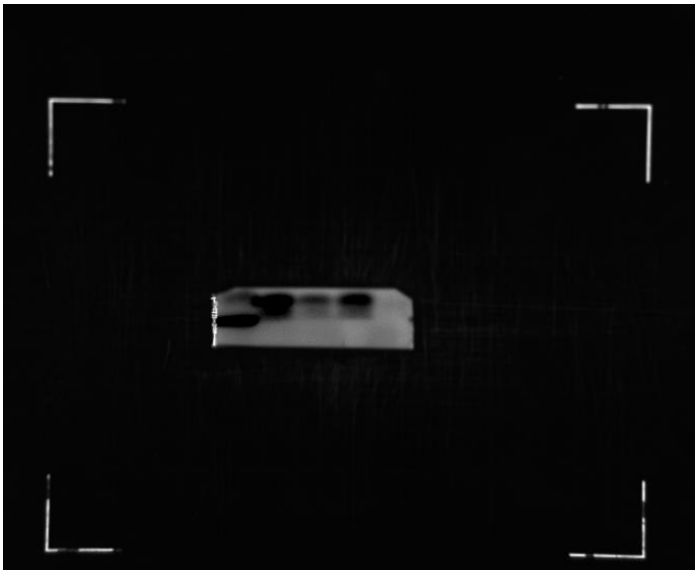

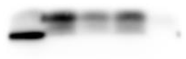

Figure 2C

Ev and OE actin

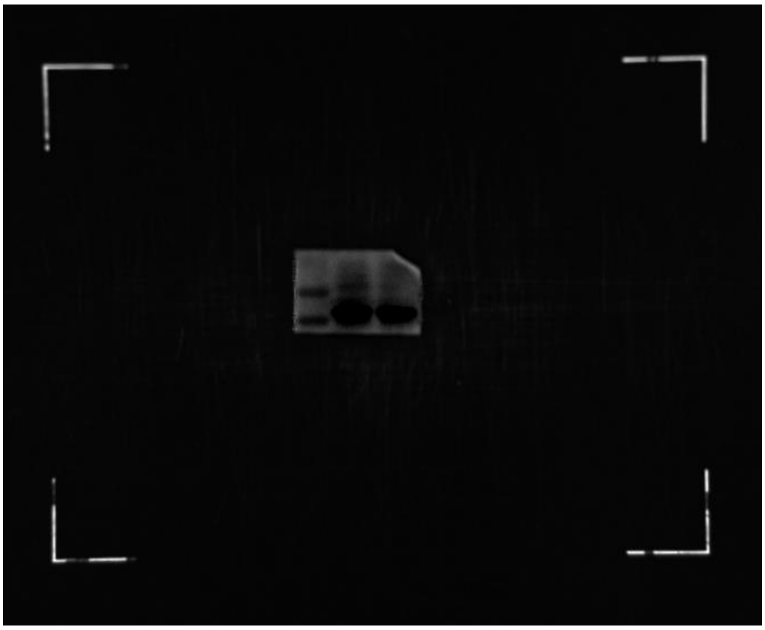

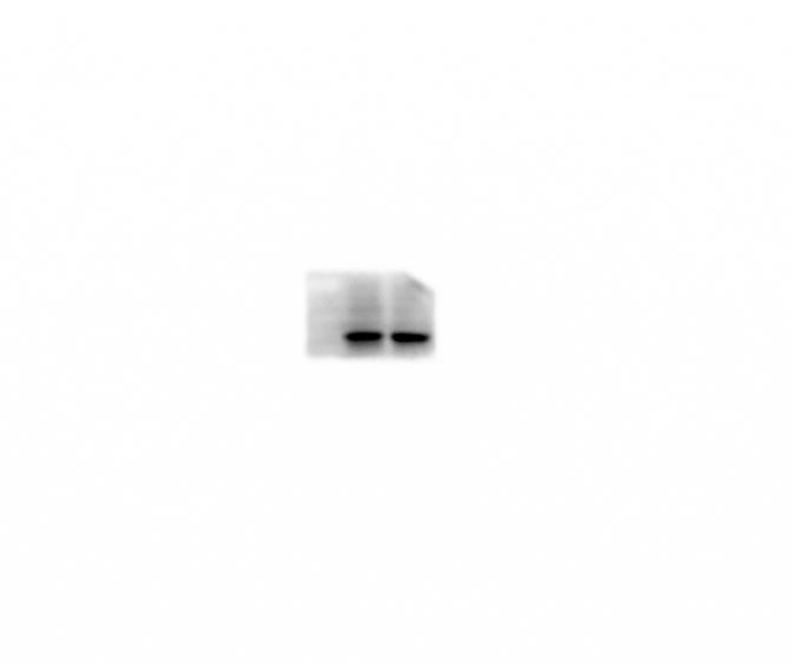

Ev and OE CSTB

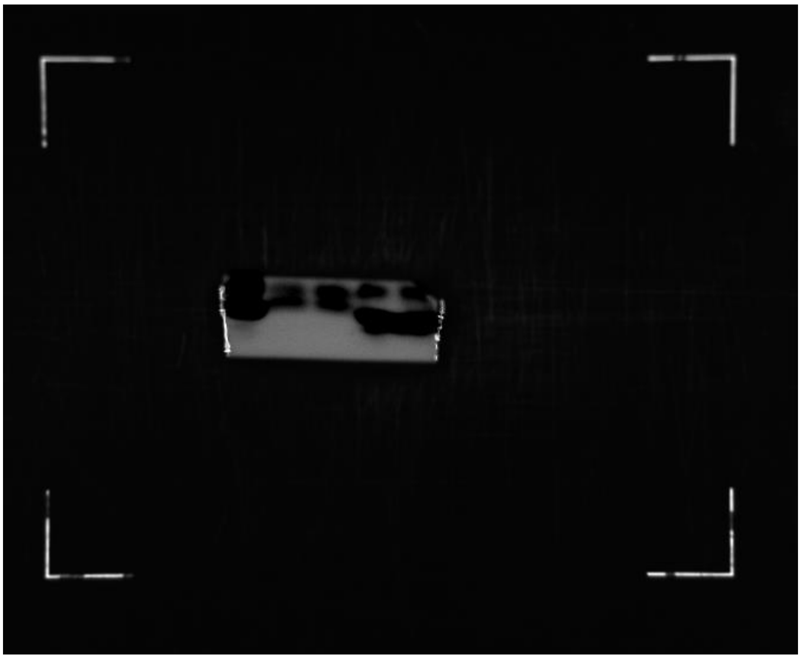

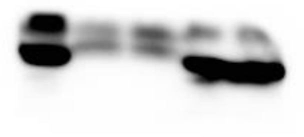

Figure 1H

Tissue actin

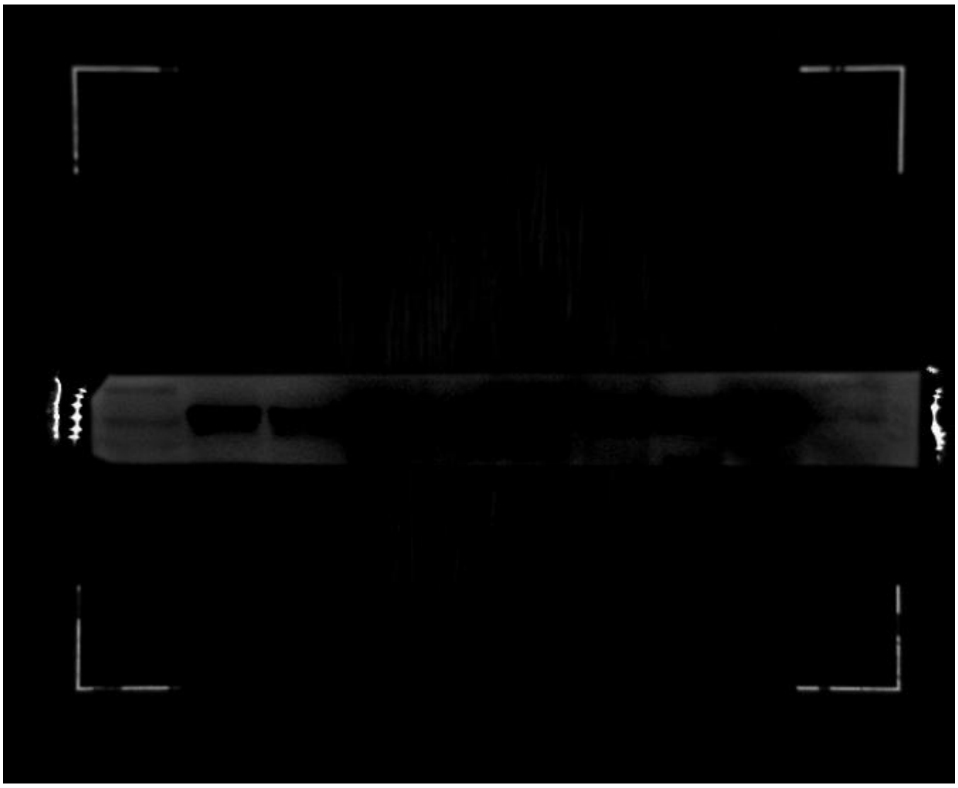

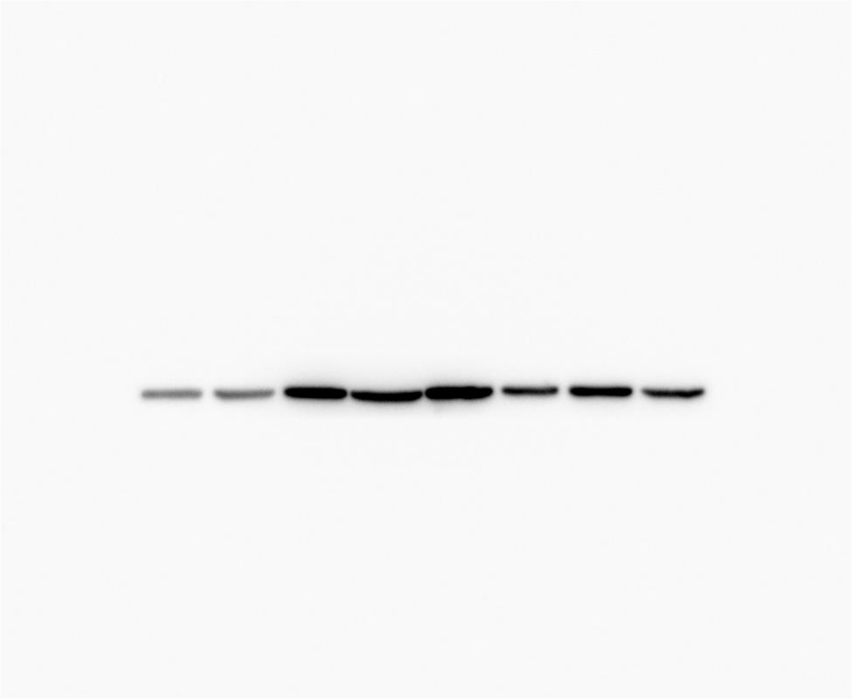

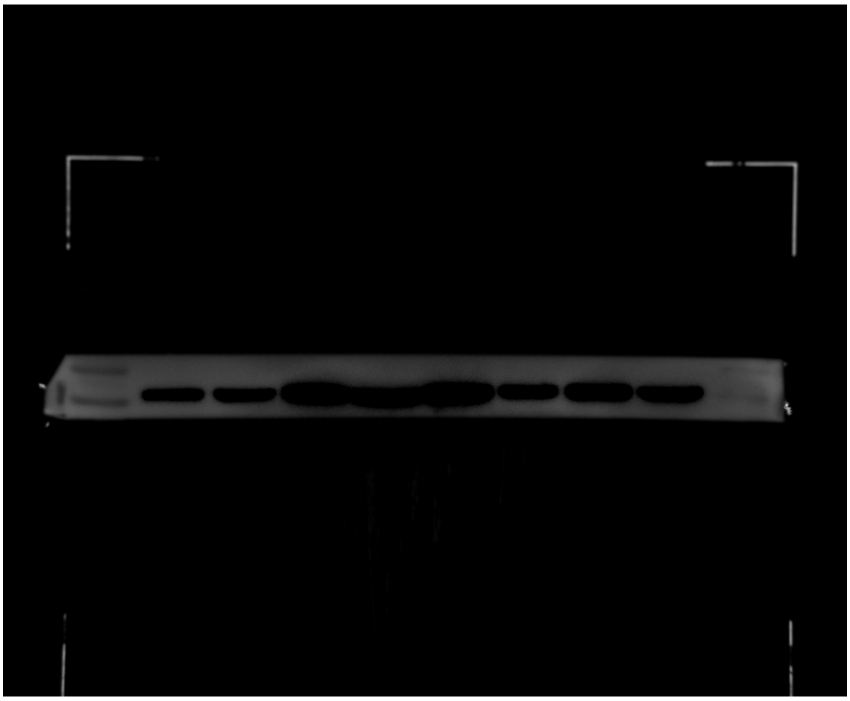

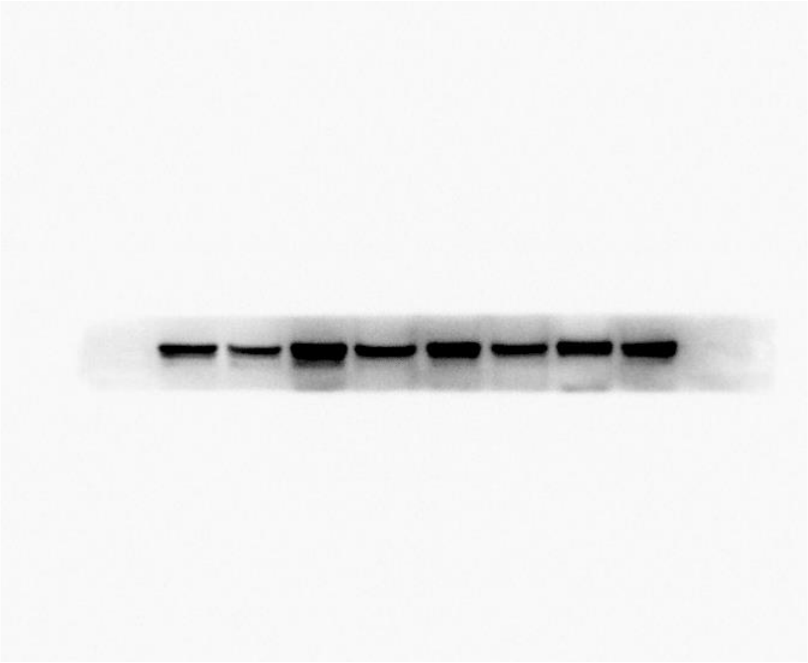

Tissue CSTB

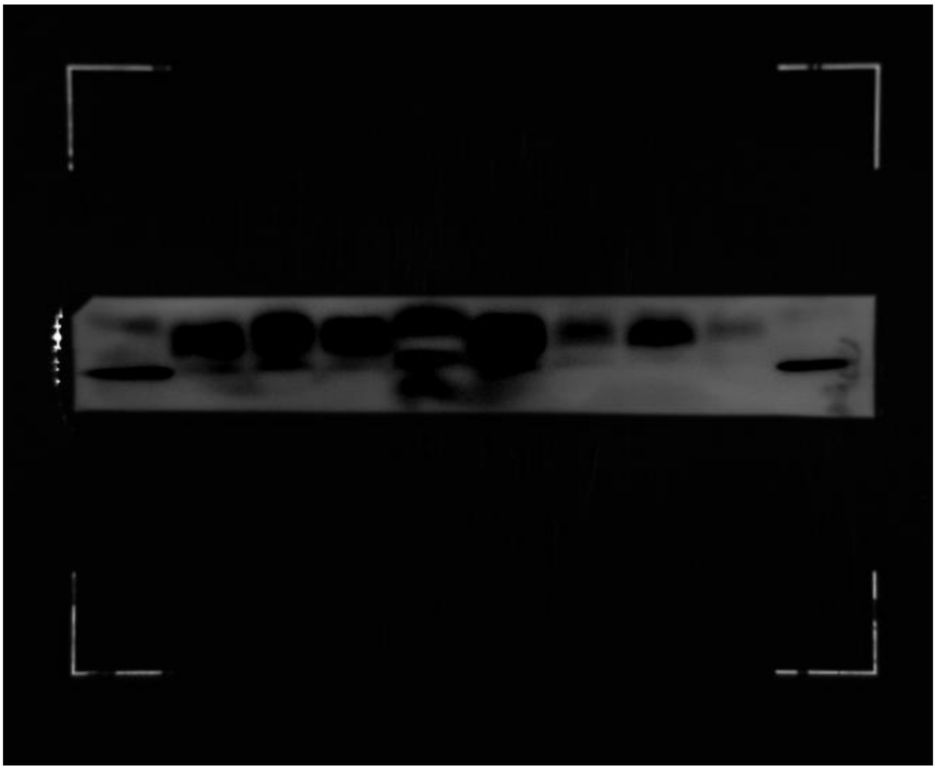

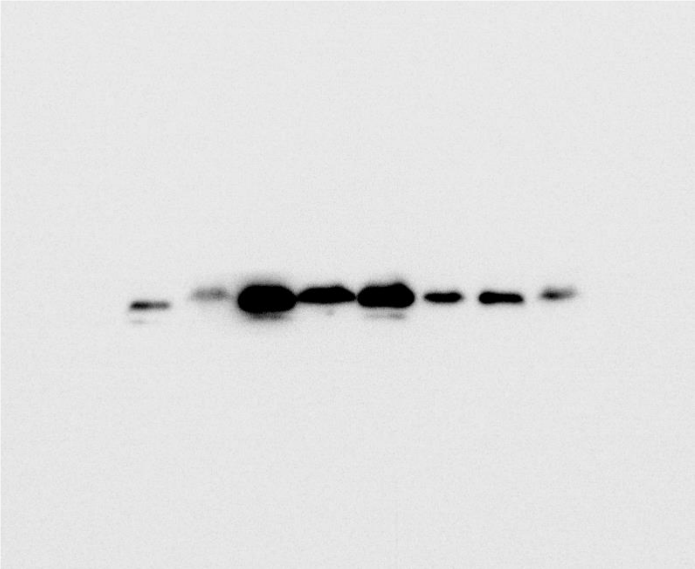

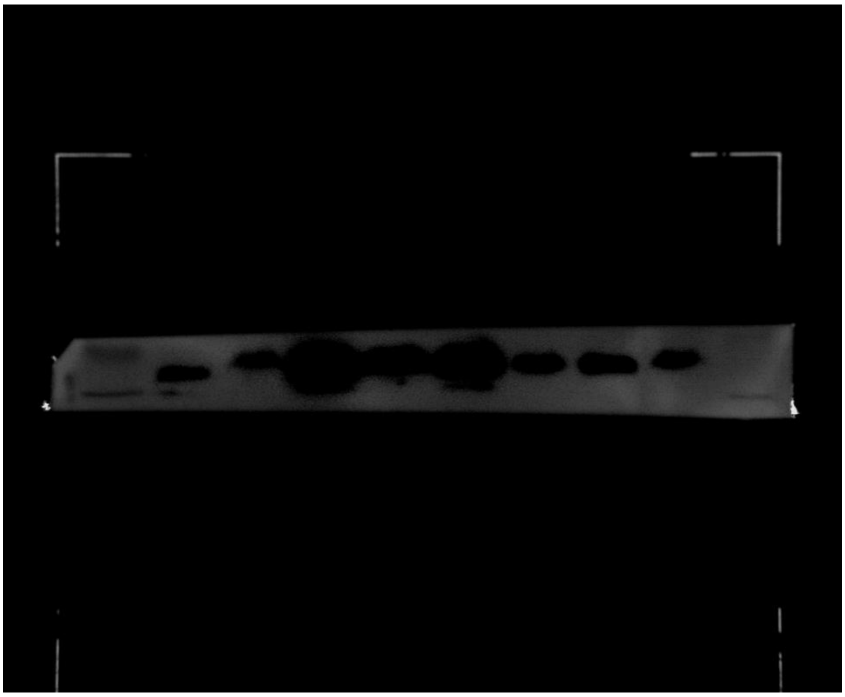

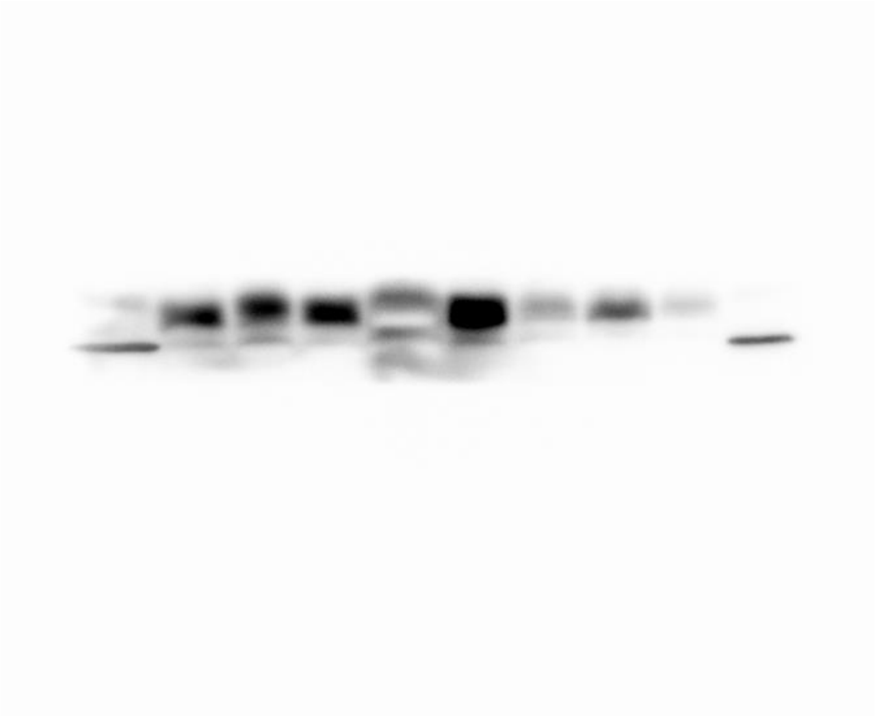

Supplement: Supplementary file 1 [file curroncol-32-00056-s001.zip › curroncol-3356729-supplementary/WB figure/WB word.pdf]
