# Supplementary figures and images for: Cystatin B Promotes the Proliferation, Migration, and Invasion of Intrahepatic Cholangiocarcinoma
Source: Curr Oncol. 2025 Jan 21;32(2):56. doi: 10.3390/curroncol32020056 (PMC11854580; doi:10.3390/curroncol32020056)

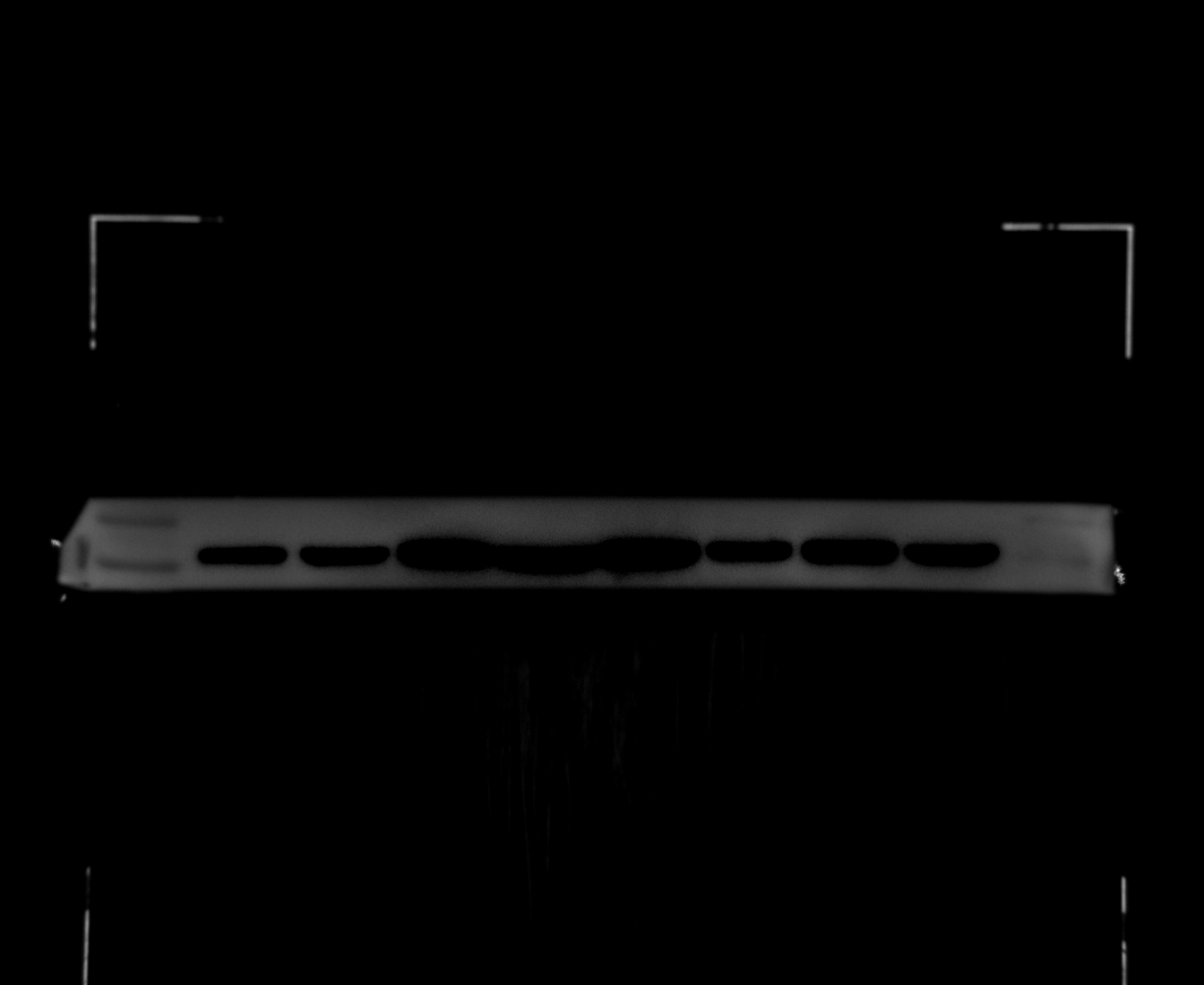

Supplement: Supplementary file 1 [file curroncol-32-00056-s001.zip › curroncol-3356729-supplementary/WB figure/figure1H/ACTIN_1_240523_150905_00.01.000_0_4000.tif]

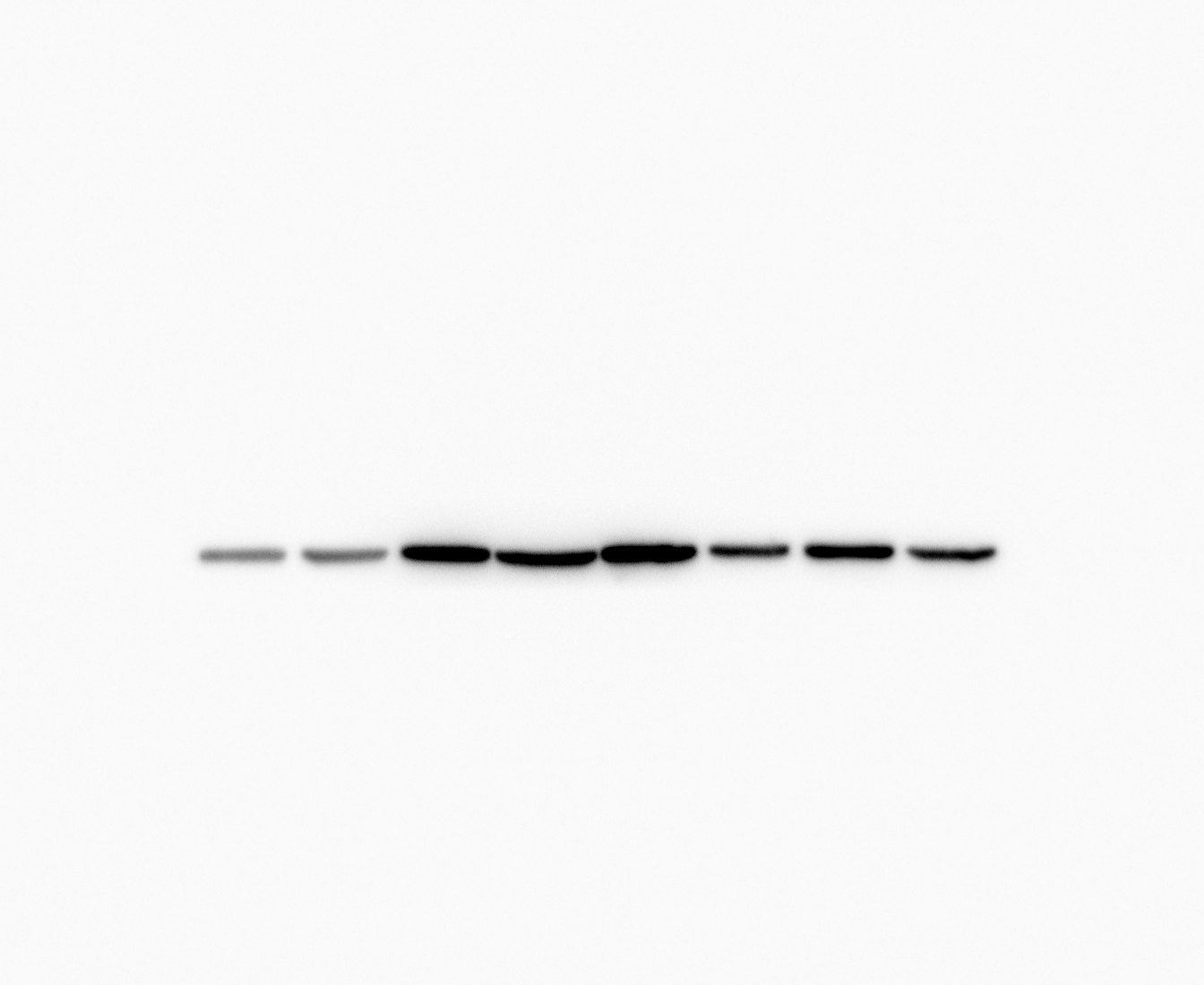

Supplement: Supplementary file 1 [file curroncol-32-00056-s001.zip › curroncol-3356729-supplementary/WB figure/figure1H/ACTIN_1_240523_150905_00.01.000_2_6000.tif]

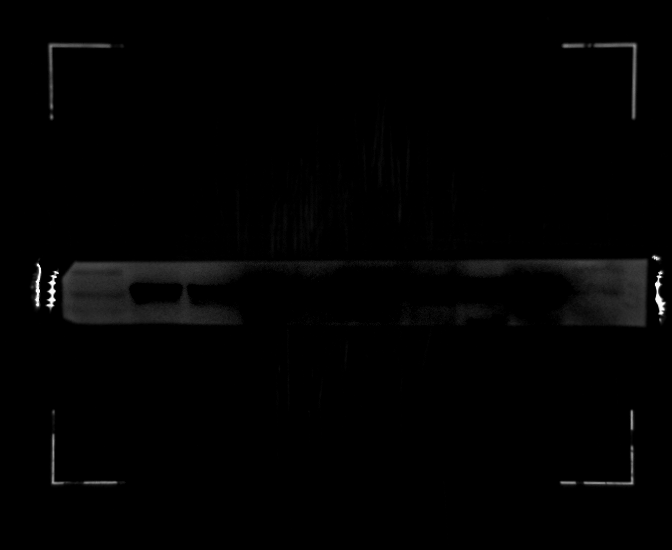

Supplement: Supplementary file 1 [file curroncol-32-00056-s001.zip › curroncol-3356729-supplementary/WB figure/figure1H/ACTIN_3_240315_205344_00.01.000_0_4796.tif]

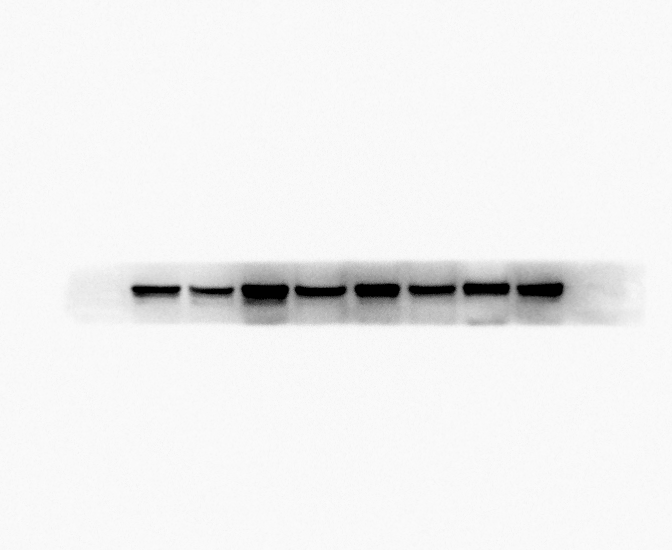

Supplement: Supplementary file 1 [file curroncol-32-00056-s001.zip › curroncol-3356729-supplementary/WB figure/figure1H/ACTIN_3_240315_205344_00.01.000_1_4796.tif]

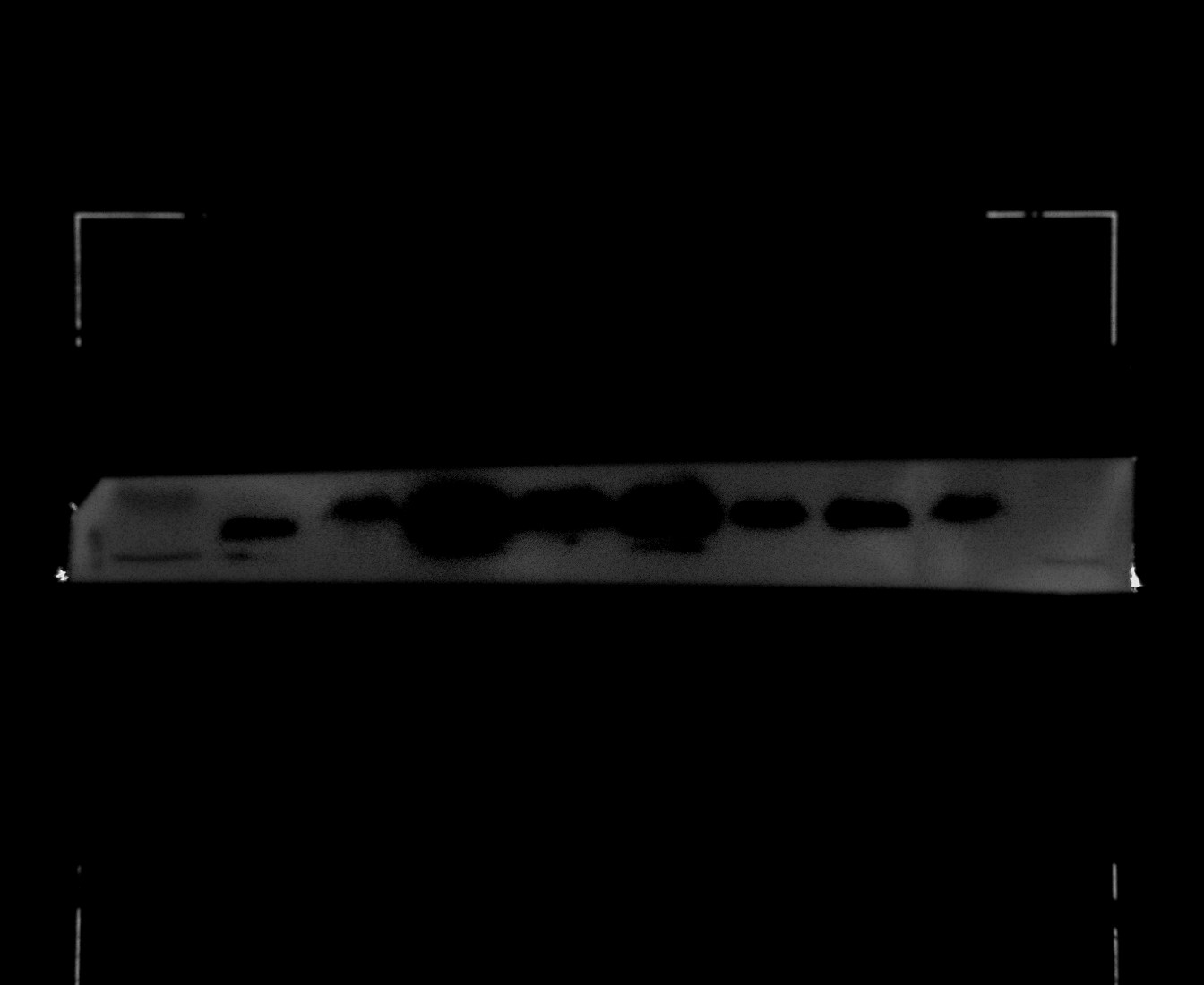

Supplement: Supplementary file 1 [file curroncol-32-00056-s001.zip › curroncol-3356729-supplementary/WB figure/figure1H/CSTB_1_240523_151113_00.05.000_0_1000.tif]

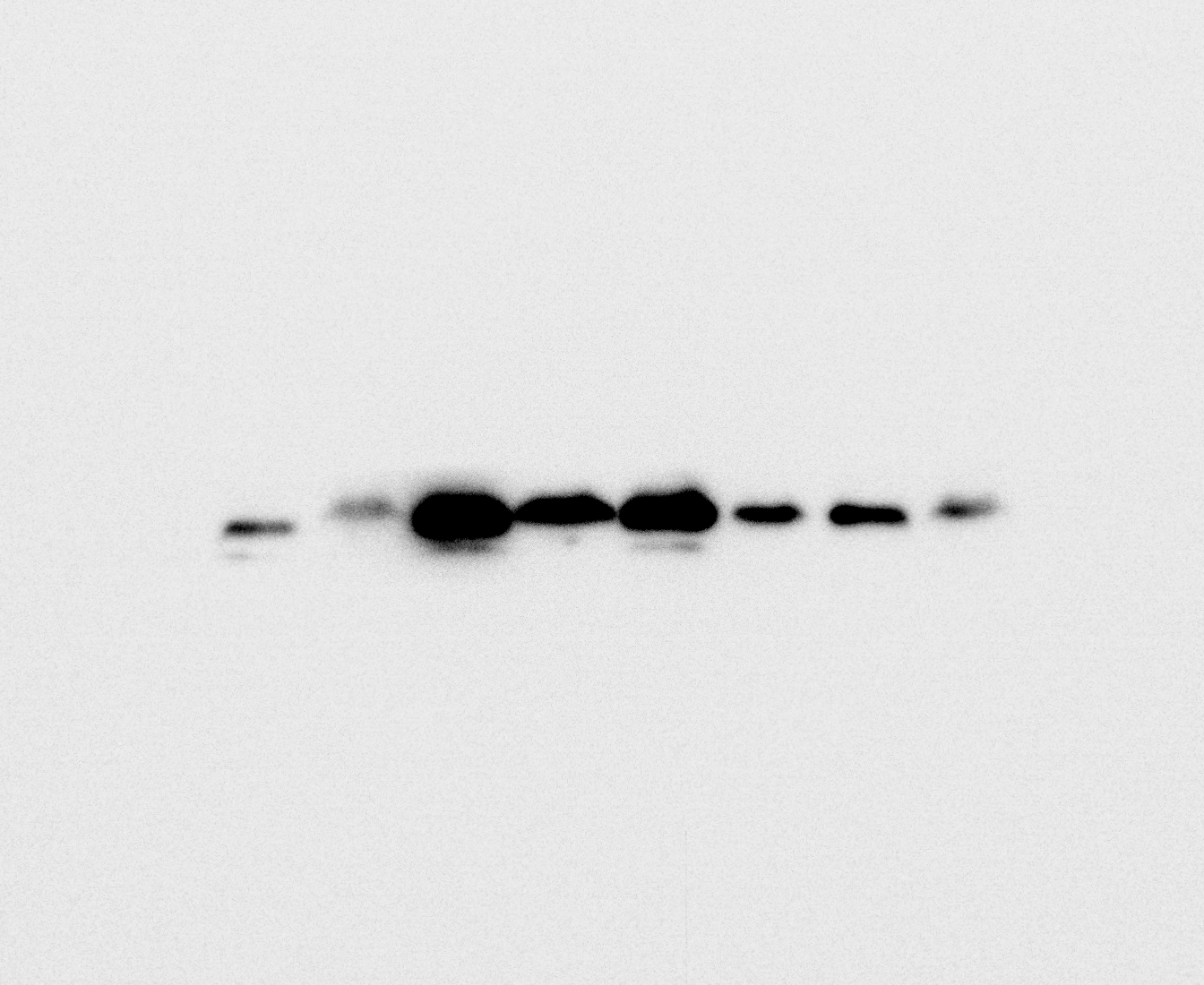

Supplement: Supplementary file 1 [file curroncol-32-00056-s001.zip › curroncol-3356729-supplementary/WB figure/figure1H/CSTB_1_240523_151113_00.05.000_1_1000.tif]

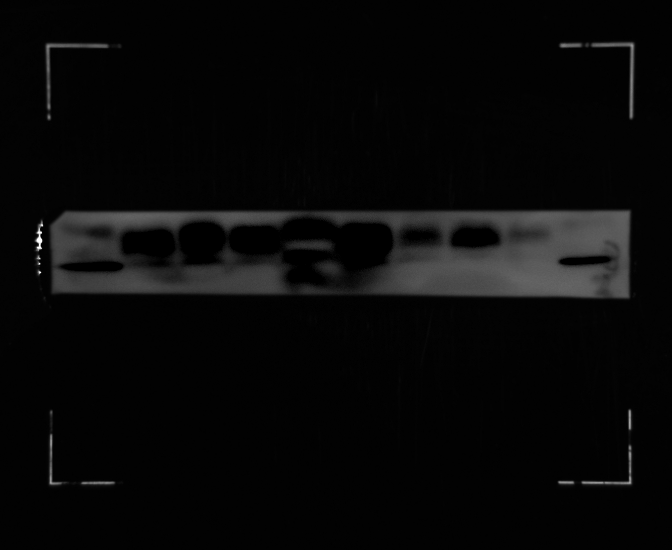

Supplement: Supplementary file 1 [file curroncol-32-00056-s001.zip › curroncol-3356729-supplementary/WB figure/figure1H/CSTB_3_240315_211511_00.02.000_0_15994.tif]

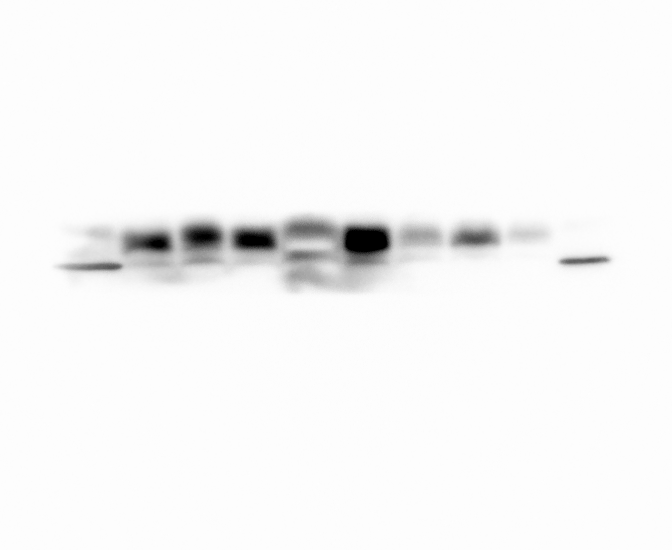

Supplement: Supplementary file 1 [file curroncol-32-00056-s001.zip › curroncol-3356729-supplementary/WB figure/figure1H/CSTB_3_240315_211511_00.02.000_1_15994.tif]

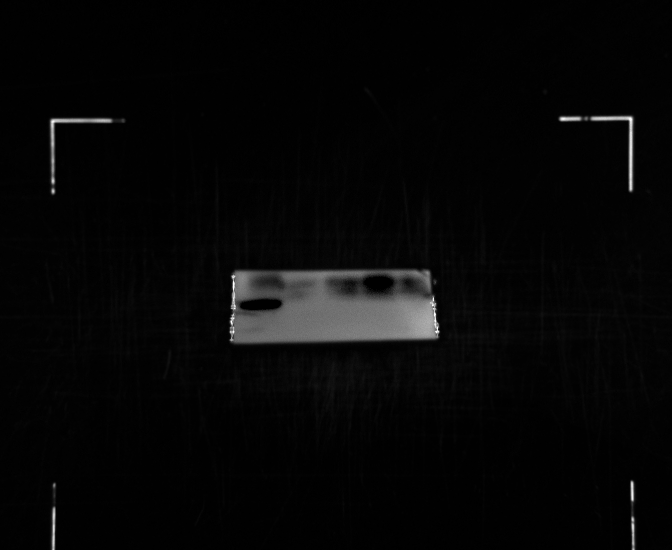

Supplement: Supplementary file 1 [file curroncol-32-00056-s001.zip › curroncol-3356729-supplementary/WB figure/figure2A/YESHENG_CSTB_231225_162200_00.02.000_0_14833.tif]

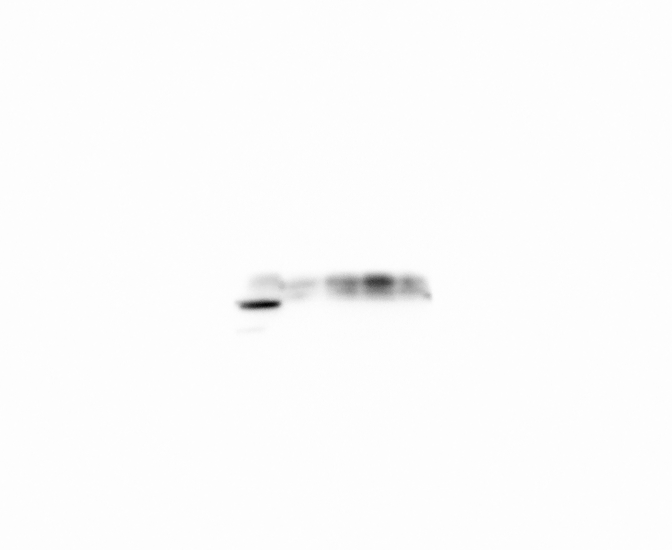

Supplement: Supplementary file 1 [file curroncol-32-00056-s001.zip › curroncol-3356729-supplementary/WB figure/figure2A/YESHENG_CSTB_231225_162200_00.02.000_1_14833.tif]

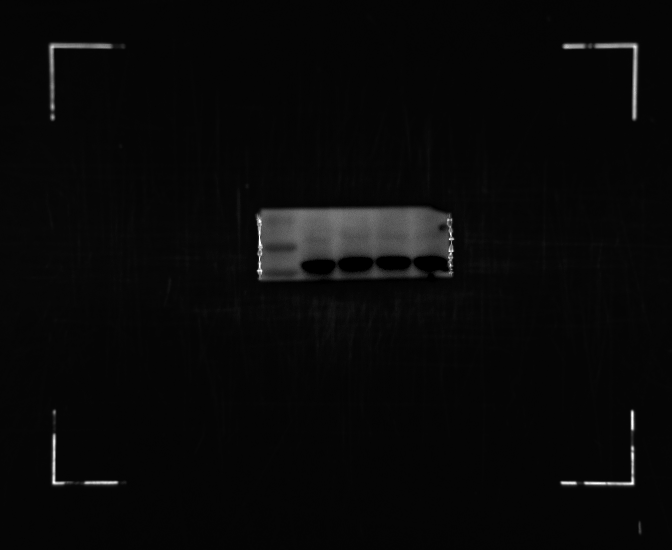

Supplement: Supplementary file 1 [file curroncol-32-00056-s001.zip › curroncol-3356729-supplementary/WB figure/figure2A/野生ACTIN(CSTBNC676869_ACTIN_231225_162934_00.12.205_0_37085).tif]

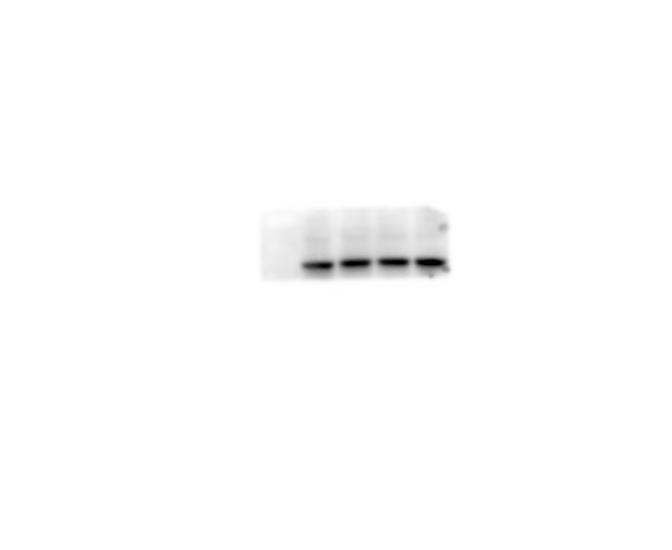

Supplement: Supplementary file 1 [file curroncol-32-00056-s001.zip › curroncol-3356729-supplementary/WB figure/figure2A/野生ACTIN(CSTBNC676869_ACTIN_231225_162934_00.12.205_2_44170).tif]

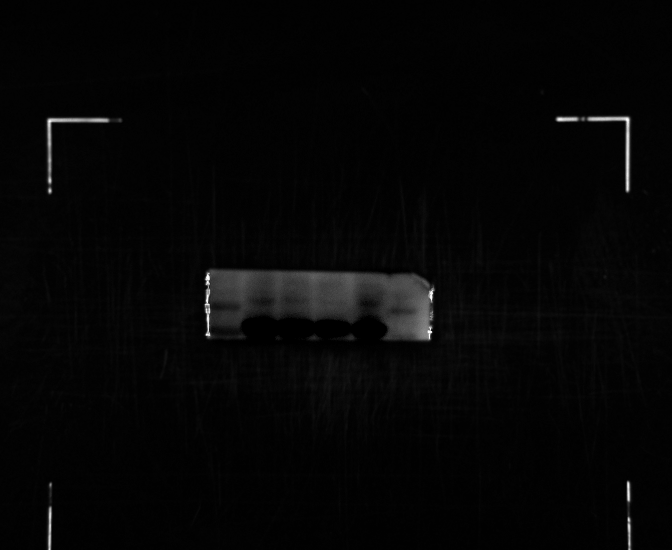

Supplement: Supplementary file 1 [file curroncol-32-00056-s001.zip › curroncol-3356729-supplementary/WB figure/figure2B/CSTBNC676869_ACTIN_2_231225_163407_00.12.205_0_20657.tif]

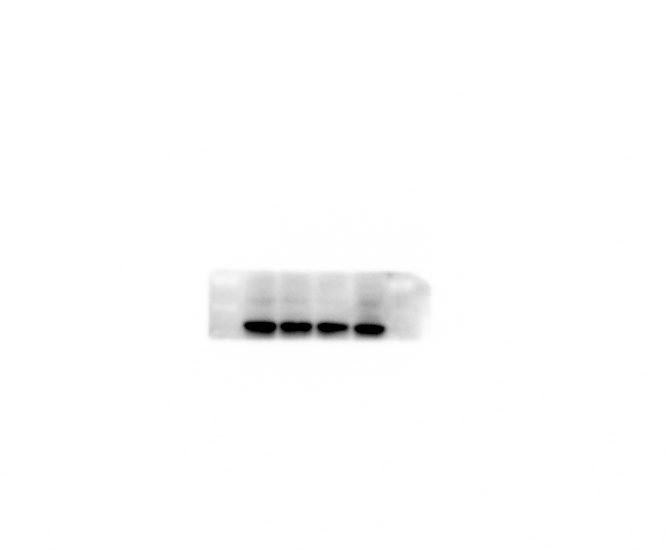

Supplement: Supplementary file 1 [file curroncol-32-00056-s001.zip › curroncol-3356729-supplementary/WB figure/figure2B/CSTBNC676869_ACTIN_2_231225_163407_00.12.205_1_23657.tif]

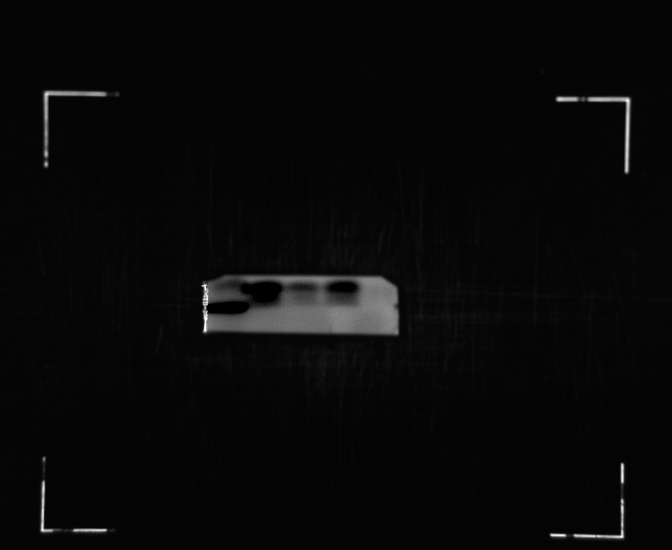

Supplement: Supplementary file 1 [file curroncol-32-00056-s001.zip › curroncol-3356729-supplementary/WB figure/figure2B/CSTBNC676869_CSTB_2_231225_164214_00.12.205_0_65204.tif]

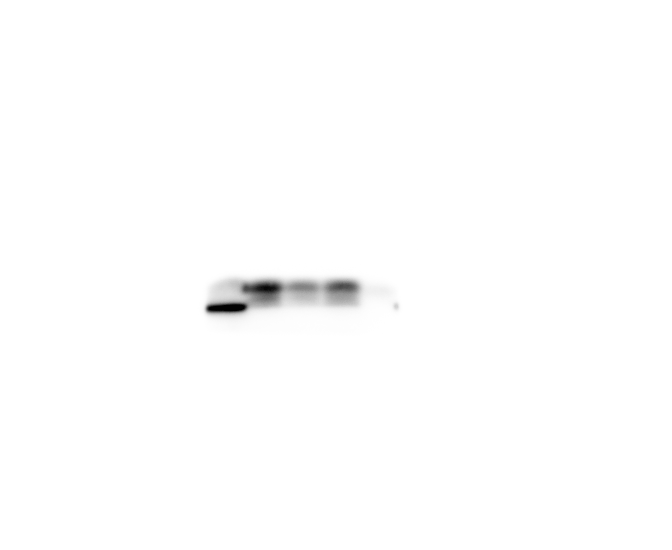

Supplement: Supplementary file 1 [file curroncol-32-00056-s001.zip › curroncol-3356729-supplementary/WB figure/figure2B/CSTBNC676869_CSTB_2_231225_164214_00.12.205_3_236816.tif]

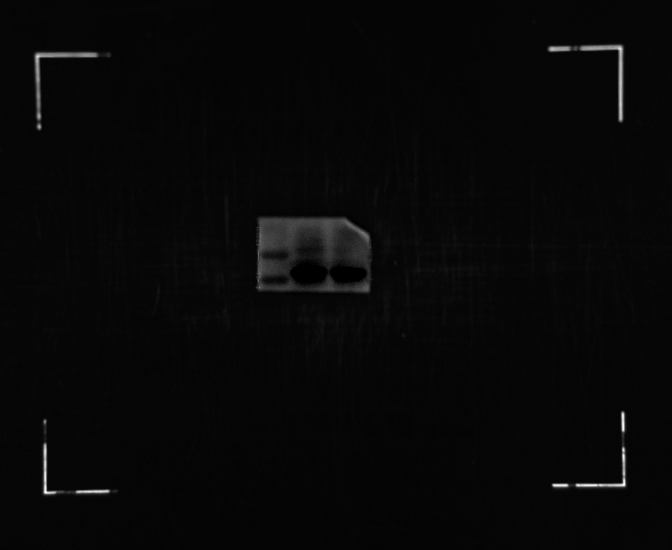

Supplement: Supplementary file 1 [file curroncol-32-00056-s001.zip › curroncol-3356729-supplementary/WB figure/figure2C/RBENCOE_ACTIN_2_231225_165446_00.12.205_0_22333.tif]

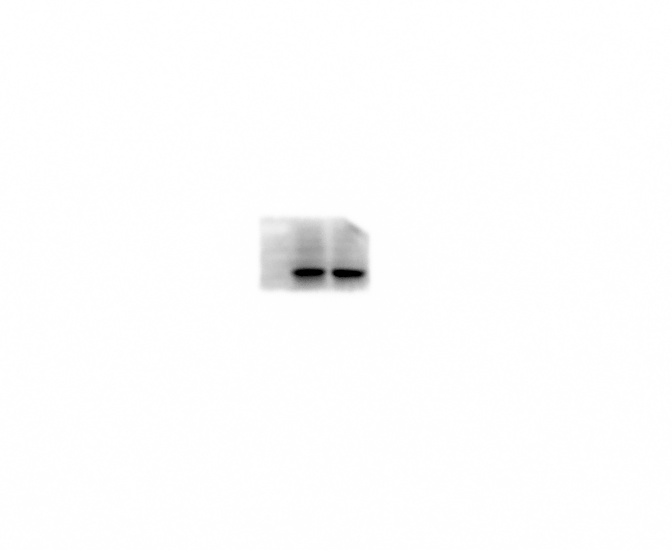

Supplement: Supplementary file 1 [file curroncol-32-00056-s001.zip › curroncol-3356729-supplementary/WB figure/figure2C/RBENCOE_ACTIN_2_231225_165446_00.12.205_1_22333.tif]

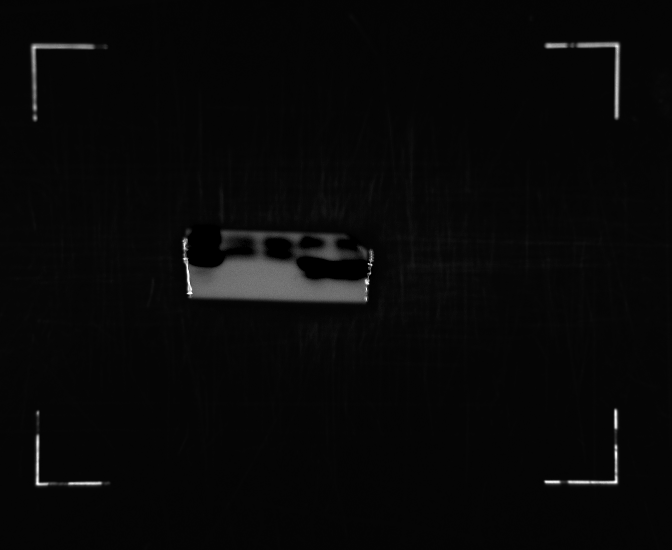

Supplement: Supplementary file 1 [file curroncol-32-00056-s001.zip › curroncol-3356729-supplementary/WB figure/figure2C/RBENCOE_CSTB_231225_160107_01.14.000_0_65535.tif]

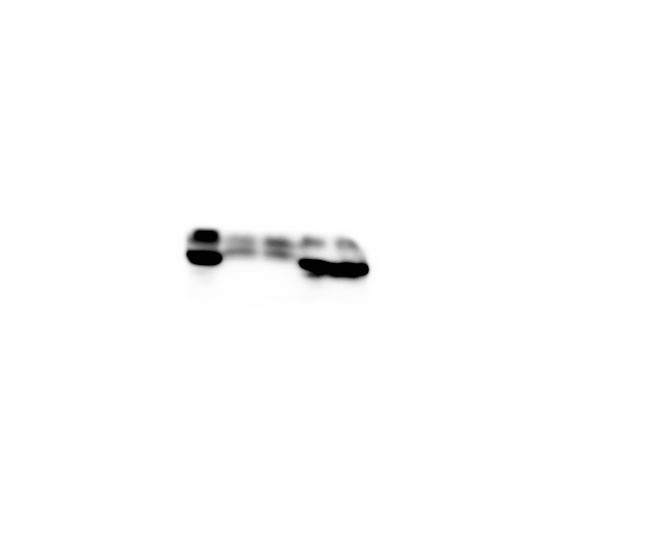

Supplement: Supplementary file 1 [file curroncol-32-00056-s001.zip › curroncol-3356729-supplementary/WB figure/figure2C/RBENCOE_CSTB_231225_160107_01.14.000_1_65535.tif]
